# Multimodality cardiac imaging of a ventricular septal rupture post myocardial infarction: a case report

**DOI:** 10.1186/1756-0500-5-583

**Published:** 2012-10-25

**Authors:** Surinder Dhaliwal, Robin Ducas, Liu Shuangbo, David Horne, John Lee, Farrukh Hussain, Iain DC Kirkpatrick, Davinder S Jassal

**Affiliations:** 1 Department of Radiology, University of Manitoba, Winnipeg, Manitoba, Canada; 2 Section of Cardiology, Department of Internal Medicine, University of Manitoba, Winnipeg, Manitoba, Canada; 3 Section of Internal Medicine, Department of Internal Medicine, University of Manitoba, Winnipeg, Manitoba, Canada; 4 Section of Cardiac Surgery, Department of Surgery, University of Manitoba, Winnipeg, Manitoba, Canada; 5 Institute of Cardiovascular Sciences, St. Boniface Research Centre, University of Manitoba, Winnipeg, Manitoba, Canada

**Keywords:** Echocardiography, Cardiac Mri, Ventricular septal rupture

## Abstract

**Background:**

Ventricular septal rupture (VSR), a mechanical complication following an acute myocardial infarction (MI), is thought to result from coagulation necrosis due to lack of collateral reperfusion. Although the gold standard test to confirm left-to-right shunting between ventricular cavities remains invasive ventriculography, two-dimensional transthoracic echocardiography (TTE) with color flow Doppler and cardiac MRI (CMR) are reliable tests for the non-invasive diagnosis of VSR.

**Case presentation:**

A 62-year-old Caucasian female presented with a late case of a VSR post inferior MI diagnosed by multimodality cardiac imaging including TTE, CMR and ventriculography.

**Conclusion:**

We review the presentation, diagnosis and management of VSR post MI.

## Background

Ventricular septal rupture (VSR), a mechanical complication following an acute myocardial infarction (MI), is thought to result from coagulation necrosis due to lack of collateral reperfusion. Although the gold standard test to confirm left-to-right shunting between ventricular cavities remains invasive ventriculography, two-dimensional transthoracic echocardiography (TTE) with color flow Doppler and cardiac MRI (CMR) are reliable tests for the non-invasive diagnosis of VSR. Although there are several case reports of VSR post MI, our case illustrates the use of multimodality cardiac imaging for the complete delineation of this mechanical complication prior to surgical repair.

## Case presentation

**Figure 1:**
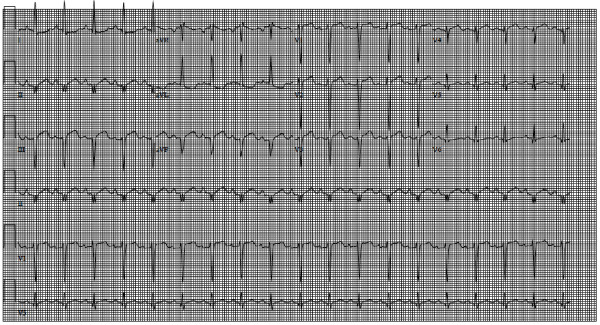
**A 12 lead EKG demonstrating sinus tachycardia with Q waves in leads II**, **III and AVF.**

**Figure 2:**
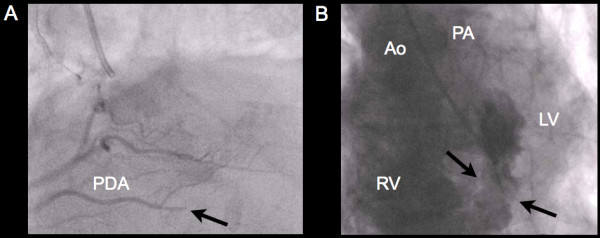
**A)A cardiac catheterization demonstrating complete occlusion of the distal PDA(arrow);B)Large basal**-**mid inferoseptal ventricular septal rupture with left to right shunting on left ventriculography.** PDA, posterior descending artery; Ao, aorta; RV, right ventricle; PA, pulmonary artery; LV, left ventricle.

**Figure 3:**
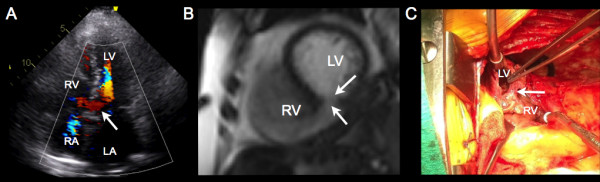
**A) An apical 4 chamber view on TTE demonstrating left to right shunting across the interventricular septum on color Doppler (arrow); B) Short axis balanced steady-state free precession CMR image demonstrates rupture of the mid inferior septal segment (arrows) with free communication between the right (RV) and left (LV) ventricles; C) A trans-ventricular exposure of the diaphragmatic surface through the infarcted myocardium at the time of surgery.** The infarct incision on the diaphragmatic surface of the heart reveals the VSR, with a free edge (arrow) that has already matured. RA, right atrium; LA, left atrium; RV, right ventricle; LV, left ventricle.

A 62-year-old Caucasian female presented with a one week history of generalized weakness and shortness of breath on exertion. Clinical assessment revealed a new grade III/VI pansystolic murmur at the left lower sternal border, elevated cardiac biomarkers with a high sensitivity troponin T of 990 ng/L, and electrocardiographic evidence of sinus tachycardia with Q waves in the inferior leads (Figure 1). Chest radiograph at presentation was within normal limits with no evidence of acute pulmonary edema. Cardiac catheterization demonstrated complete occlusion of the distal posterior descending artery, with evidence of a left ventricular septal rupture (VSR) (Figure 2, Additional file 1: Video 1). Transthoracic echocardiography (TTE) demonstrated hypokinesis of the basal inferior wall of the left ventricle (LV) with left to right shunting across the interventricular septum on color Doppler (Figure 3A). Cardiac MRI (CMR) demonstrated a VSR in the mid inferior septal segment of the LV with a Qp/Qs shunt of 4.2 (Figure 3B, Additional file 2: Video 2). A posterior VSR with mature edges was identified near the LV apex in the region of the infarction at the time of surgery (Figure 3C). Under cardio-pulmonary bypass, primary repair of the VSR was done using multiple pledgeted sutures followed by a second layer closure with Teflon felt strips.

## Discussion

Ventricular septal rupture (VSR) is a devastating complication affecting less than 1% of patients with the majority of cases occurring 3 to 7 days post MI [1]. Risk factors for the development of a VSR include hypertension, advanced age (>60), and a non-smoking status with women more commonly affected than men. Patients with a first time infarction and single vessel coronary disease, particularly involving the left anterior descending coronary artery, are at increased risk of VSR [2]. Clinically, VSR should be suspected in patients with the onset of a new systolic murmur after a documented myocardial infarction. Up to one half of affected patients may experience chest pain, while congestive heart failure (CHF) and cardiogenic shock may also develop.

Multimodality cardiac imaging using ventriculography, TTE and CMR can provide complementary information for accurate and complete delineation of the VSR. Although the gold standard test to confirm left-to-right shunting between ventricular cavities remains invasive ventriculography, it is difficult to define the exact size and morphology of the VSR. Two-dimensional TTE with color flow Doppler is a reliable test for the non-invasive confirmation of VSR by characterizing its exact location and size as well as the direction of the shunt [3]. Similar to TTE, CMR with its higher spatial resolution can accurately delineate the anatomy, location, and size of the VSR prior to surgical correction. However, CMR can also noninvasively quantify the shunt size across the interventricular septum and allows for accurate characterization of the peri-infarct zone. This is the first case report in the literature describing the use of all three complementary imaging modalities for defining a VSR post-MI.

Optimal management of patients with VSR remains controversial both in terms of timing and choice of intervention. Retrospective data has demonstrated a decrease in operative mortality rates in patients with a longer (>6 weeks) interval between myocardial infarction and surgical repair [4]. However, this longer time interval introduces an inherent survival bias by selecting the more stable patients. The choice of intervention varies with the clinical presentation. For patients presenting acutely in cardiogenic shock, emergent treatment is necessary. This may be in the form of immediate surgical repair or temporary mechanical circulatory support followed by definitive repair. Although percutaneous repair has been reported in older patients with smaller defects, no studies have directly compared both percutaneous and surgical approaches [5]. Elective surgery is preferred for patients who are hemodynamically stable.

## Conclusion

Our case demonstrates the development of a VSR after a delayed presentation of an inferior MI. The absence of ST elevation, presence of Q waves, and elevated troponin levels are the result of an infarct occurring in the preceding week. Multimodality cardiac imaging using echocardiography, CMR and cardiac catheterization provided unique and complementary information for the pre-operative characterization of the VSR prior to surgical intervention.

## Consent

Written informed consent was obtained from the patient for publication of this case report and accompanying images. A copy of the written consent is available for review by the Editor-in-Chief of this journal.

## Competing interests

The authors declare that they have no competing interests.

## Authors’ contributions

SD, RD, SL, DH, JL, FH, IK and DJ contributed to the writing of the manuscript. All authors read and approved the final manuscript.

## Supplementary Material

Additional file 1 **Video 1.** Large basal-mid inferoseptal VSR with left to right shunting on left ventriculography. Click here for file

Additional file 2 **Video 2.** Short axis balanced steady-state free precession CMR image demonstrates rupture of the mid inferior septal segment with free communication between the right (RV) and left (LV) ventricles. Click here for file
